# Microbial transformation of soil organic matter under varying agricultural management systems in Ukraine

**DOI:** 10.3389/fmicb.2023.1287701

**Published:** 2024-01-11

**Authors:** Lyudmyla Symochko, Olena Demyanyuk, Vlad Crisan, Lucian Dinca

**Affiliations:** ^1^Faculty of Biology, Uzhhorod National University, Uzhhorod, Ukraine; ^2^Department of Life Sciences, Faculty of Science and Technology, University of Coimbra, Coimbra, Portugal; ^3^Institute of Agroecology and Environmental Management, Kyiv, Ukraine; ^4^Romanian National Institute of Research and Development in Forestry “Marin Dracea” Brasov branch, Braşov, Romania

**Keywords:** cellulolytic microorganisms, cellulose destruction, fertilization, agricultural management, soil

## Abstract

**Introduction:**

This paper presents comparative studies on the content and structure of organic matter (OM) and the activity of microbiological cellulose destruction in three types of Ukrainian soils intensively used in agricultural production.

**Methods:**

The highest content of humus in the arable layer (4.9%), OM (410 t ha^−1^), and total carbon (30.9 mg C g^−1^ soil) was determined in chernic phaeozems, which is 2.2–2.5 times higher than in albic retisols. The soil of natural ecosystems is characterised by a high content of microbial carbon (C_mic_) in the carbon fraction of organic soil compounds.

**Results and discussion:**

In arable soils, the content and reserves of humus and soil organic matter (SOM) have decreased by an average of 1.5–2 times. The most considerable loss of humus reserves in the soil profile was identified in albic retisols (1.96–1.44 times) and the smallest in chernic phaeozems (1.27–1.81 times). During the long-term systematic application of mineral fertilisers, the Corg content decreased by 8-21% in chernic phaeozems, 12-33% in greyzemic phaeozems, and 6–38% in albic retisols. A significant difference of 2.1–8.0 times was determined regarding the number of aerobic cellulolytic microorganisms and 1.3–3.3 times in the potential cellulolytic activity of the studied soils. The high number of cellulose-destroying microorganisms is characteristic of chernic phaeozems with a high content of OM in the soil; the advantage over other types of studied soils was 1.4 times and 7.8 times for greyzemic phaeozems and albic retisols, respectively. Among the studied soil types, high values of CO_2_ emissions were identified in chernic phaeozems. Intensive agricultural practices in Ukrainian soils have significantly altered the content and composition of organic matter, leading to reduced humus and soil organic matter reserves. The study also underscores the importance of considering the abundance of cellulose-destroying microorganisms and their potential activity in assessing soil health and sustainability.

## 1 Introduction

Soil is a fundamental component of terrestrial ecosystems and is vital in ensuring their overall health. It provides a range of ecosystem services and is essential for food production. Moreover, soil plays a significant role in the cycling of elements within ecosystems. It serves as a sink and source for various elements, such as carbon (C), nitrogen, and phosphorus. These elements are essential for the functioning of biological processes and are recycled through soil microorganisms, plants, and other organisms in the ecosystem (Gomiero, 2016; Demyanyuk et al., 2020; Gomes et al., 2023; Symochko et al., 2023). In particular, soil plays a pivotal role in the destructive chain of the C cycle and its sequestration (or deposition), thereby influencing climate change. That is, soil has two mutually opposing functions and can act as a C sink or a C source, as soil organic matter (SOM) is in a complex balance. This balance depends on the rate of entry of organic matter (OM) into the soil and the rate of its mineralisation. Therefore, as SOM is one of the largest C sinks on Earth (O’Rourke et al., 2015), it is essential to study the influence of various environmental and anthropogenic factors on SOM, its content, accumulation, and destruction (Lehmann and Kleber, 2015; Demyanyuk et al., 2019; Fazi et al., 2019; Rodrigues et al., 2023).

Soil organic matter is an important indicator of soil quality and biological activity in agro-ecosystems. It further determines the presence of biophilic nutrients and trophic regime, soil structure, water regime, and erosion resistance. Considering this, many human activities threaten ecosystems and cause land degradation, such as desertification, loss of biodiversity, disruption of aggregates, and loss of OM and nutrients. On a global scale, the change in land use that has the greatest impact on the C cycle is related to the expansion and intensification of agriculture. The loss of SOM due to the continuous intensive cultivation of crops is a growing problem in the world (Trivedi et al., 2016; Sainju et al., 2018; Symochko, 2020). The main part of the carbon dioxide (CO_2_) released into the atmosphere is formed due to the transformation processes of organic substances in the soil. CO_2_ emissions from soil are a natural byproduct of biological processes and soil characteristics. Besides generating CO_2_, the decomposition of soil organic matter (SOM) serves as a source of metabolic energy for microorganisms and supplies plants with accessible mineral nutrients. The extent of CO_2_ emissions from the soil surface serves as a comprehensive measure of its biological activity, reflecting the intensity of destructive processes and indicating soil fertility (Weissert et al., 2016; Demyanyuk et al., 2020). Any changes in the type or amount of OM entering the soil can directly affect soil enzymatic activity, microbial biomass and activity, microbial community, or functions performed by different microbial groups (Shen et al., 2016; Chen et al., 2022). The direct and indirect effect of various agricultural practices, in particular agrochemicals, on the biological activity of the soil is manifested in the reduction of the number and biodiversity of microorganisms, the proliferation of beneficial soil microorganisms and their biotransformation, the reduction of biological nitrogen fixation, and the slowing down or activation of OM mineralisation processes (Malik et al., 2017; Litvinova et al., 2023). Microorganisms participate in numerous processes, such as the transformation of C and N. For example, as a result of their metabolic processes, microorganism excretions help in soil structure formation and maintaining productivity and quality of the environment (Bhagat et al., 2018; Agumas et al., 2020). Furthermore, soil microorganisms largely mediate the flow of C through agricultural ecosystems, and microbial communities can quickly respond to environmental changes by changing the biomass and species composition and structure of the microbiocenosis (Wang et al., 2018; Symochko et al., 2021a).

Since biological indicators of soil quickly respond to natural and anthropogenic factors, they are widely used in monitoring studies, to assess soil health, and to develop environmentally safe agricultural technologies (Rinot et al., 2019; Symochko et al., 2021b,^2023^; Bhaduri et al., 2022).

In the biosphere, the most common organic compound and the primary source of C is cellulose, which is also considered an important global producer of renewable resources (Feng et al., 2007; Adewuyi, 2022). In soils, cellulose accumulates mainly from plant residues since it is the main component of plant cell walls. To a lesser degree, it is synthesised in small amounts by soil micromycetes and bacteria (Lynd et al., 2002). Microbial mechanisms controlling the degradation of SOM, particularly the production of cellulose biopolymers, remain a critical knowledge gap in understanding and modelling the terrestrial C cycle. In addition, it has been shown that soil bacterial communities and genes differ depending on land management and soil microenvironment (Lynd et al., 2002). The activity of cellulases and the process of cellulose destruction in the soil of agro-ecosystems is determined by several factors, including temperature, pH of the environment, soil structure, chemical structure of OM and its distribution according to the soil profile, and the quality of introduced organic substances with fertilisers (Choi et al., 2018). Consequently, considering these factors makes it possible to use cellulolytic activity as a sensitive test for assessing the state of the soil under the influence of various agricultural technologies and pollution by heavy metals (Fan et al., 2012; Krzyśko-Łupicka et al., 2017). Therefore, this work aimed to investigate the content of OM in various soil types of Ukraine in natural and agricultural ecosystems and the processes of microbial destruction of cellulose biopolymer.

## 2 Materials and methods

### 2.1 Experimental site and investigation design

The study was conducted in the Laboratory of Microorganism Ecology of the Institute of Agroecology and Environmental Management of the National Academy of Agrarian Sciences of Ukraine. The initial data for the analysis, calculations, and statistical analysis were the product of many years of research (2001–2021). Soils of natural ecosystems and stationary field experiments of the National Academy of Agrarian Sciences of Ukraine were studied, and three types of soils with contrasting agrochemical indicators (Table 1) in different regions of Ukraine were chosen for research.

**TABLE 1:** Agrochemical characteristic of soil in stationary field experiments, 0–20 cm.

Type of soil/geographic coordinates	pH	Humus, %	Content, mg kg^–1^ of soil		
			**Nitrogen which easily hydrolises**	**Active phosphorus**	**Exchangeable potassium**
Albic Retisols 51°7′00″ N 31°10′07″ E	4.9–5.0	1.1	74	170	68
Greyzemic Phaeozems 50°70′63″ N 26°54′77″ E	5.8–6.1	1.8	117	235	87
Chernic Phaeozems 49°75′25″ N 27°39′20″ E	5.7–6.7	3.6	185	146	85

All values are given on dry weight basis.

### 2.2 Description of sites and soil sampling

The soil sampling was done using standardised methods (ISO 10381-6, 1993; ISO 10381-2, 2002). Soil samples from each experiment variant and fallow were taken in fivefold repetitions from the upper 0–20 cm topsoil layer when the system reached its climax—a stable, equilibrium state at the end of June. All samples were prepared for analysis by drying and grinding to a size of <3 mm; visible remains of plants and mesofauna were removed. In field experiments, the effect of long-term application of different fertilisation systems was studied. Treatments included (1) a control (Con) where no fertilisers were used (control; Con), (2) application of only mineral (Min) or (3) organic fertilisers (Org), and (4) a combination of organic and mineral fertilisers (Org + Min) (Table 2).

**TABLE 2:** Fertilisation scheme in stationary field experiments at the National Academy of Agrarian Sciences Network Institutions.

Type of soil	Experimental variant (fertilisation system)*			
	**Control**	**Mineral**	**Organic**	**Organo-mineral**
Albic retisols	Without fertilisers	N_80_P_75_K_75_	Manure 20 t/ha	Manure 10 t/ha + Straw + Cover Crop + N_80_P_75_K_75_
Greyzemic phaeozems	Without fertilisers	N_60_P_68_K_63_	Manure 20 t/ha	Manure 10 t/ha + N_60_P_68_K_63_
Chernic phaeozems	Without fertilisers	N_110_P_60_K_120_	Manure 16 t/ha + straw + cover crop	Manure 8 t/ha + N_55_P_30_K_60_

*Fertiliser doses are provided based on 1 ha of area.

In agroecosystems was cultivated winter wheat.

### 2.3 Soil carbon content

The content of total carbon (C_tot_), carbon of organic compounds (C_org_) and carbon of microbial biomass (C_mic_) was determined in the soil samples. The contents of C_tot_ and C_org_ were determined by the dry combustion method (ISO 10694, 1995). The total content of humus in the soil was determined according to the method by I. Tyurin DSTU 4289:2004 (2005). Further, the stock of SOM and humus in the soil profile was calculated while also considering the density of the studied soils. Considering that OM makes up 50% of C, a factor of 0.50 was used to convert SOM to C_org_, which is more accurate for estimating soil C content based on SOM measurements (Pribyl, 2010). The content of total microbial biomass (C_mic_) in the soil was determined using the rehydration method. For this, samples were gently dried at 65–70°C for 24 h, followed by extraction with a 0.5 M0.5 M K_2_SO_4_ solution (Anderson and Domsh, 1978; Blagodatsky et al., 1987).

### 2.4 Soil cellulolytic activity

The modified Christensen method (Zviahyntsev, 1991; Alef and Nannipieri, 1995), using model systems based on filter paper degrading (FPD) assay, was used to determine the potential cellulolytic activity of the soil. The rate of cellulose decomposition was estimated by the percentage loss of mass of the substrate (standard cellulose containing 100% glucan and 0.04% ash; density 75 g/m^2^) after 30 days of exposure under optimal conditions of 25–27°C and soil moisture at 60% of the total water capacity. The soil samples weighed 40 g, and the experiment was repeated five times. This method also made it possible to visually observe the development of cellulose-destroying microorganisms by specific colour zones. The number of cellulolytic microorganisms in the soil was determined by sowing a soil suspension on Vinohradsky’s medium (Volkohon et al., 2010). A 10 g soil sample was placed in a sterile mortar, microorganisms were dispersed from the soil particles, and a 10-fold dilution of the original soil suspension was prepared. Afterward, the nutrient medium was incubated at 25–28°C for 7–14 days. Then, colonies grown on the medium were counted, assuming one colony was formed from each viable cell. Finally, the number of microorganisms was expressed in colony-forming units (CFU) per 1 g of dry soil.

### 2.5 CO_2_ emission

CO_2_ production by soil was measured in laboratory conditions (incubation time 24 h, temperature 24–25°C, soil humidity −60% of full moisture capacity) by the adsorption method (Volkohon et al., 2010). The amount of CO_2_ coming from the soil after alkaline adsorption was determined by titration with HCl solution.

### 2.6 Statistical analysis

Statistical software Statistica 10.0 (Stat Soft Inc., USA) was used to evaluate the data from the bioassays. Analyses had 3–5 replicates. In addition, mean values (x) and their standard deviations (SD) were determined. The level of significance chosen for the study was *P* < 0.05.

## 3 Results and discussion

Phaeozems cover an estimated 190 million hectares worldwide. In Ukraine, it makes up 14.9% of the territory’s total area, 10% of which is actively used for agricultural production. Therefore, it is crucial to investigate alterations in the characteristics of these soils, specifically changes in soil organic matter (SOM) content and transformation processes. This becomes even more essential when considering the influence of environmental factors, ongoing climate change, and substantial anthropogenic pressure on agricultural soils (Helevera and Topolnyi, 2018; Rodrigues et al., 2023). As seen in Tables 2–4, these studied soils have characteristic differences in their contents and reserves of humus and SOM. In chernic phaeozems (Table 3) of the natural ecosystem, the content of C_tot_ and SOM is 2.2–2.3 times higher than in albic retisols (Table 4). The higher content of SOM and C_tot_ is typical for soils of natural ecosystems (Sun et al., 2017).

**TABLE 3:** Content and reserves of organic matter and carbon pool in the chernic phaeozems of Ukraine (x ± SD, *n* = 15).

Treatment	Natural ecosystem	Agroecosystem*			
		**1**	**2**	**3**	**4**
Humus, %	4.9 ± 0.04	3.3 ± 0.01	3.4 ± 0.02	3.7 ± 0.02	3.6 ± 0.03
Humus in profile, t ha^–1^	380 ± 4.2	210 ± 2.1	231 ± 2.6	300 ± 3.1	288 ± 2.3
SOM in profile, t ha^–1^	410 ± 4.1	296 ± 2.5	312 ± 2.6	342 ± 3.0	324 ± 2.9
C_tot_, mg C g^–1^ soil, 0–20 cm	30.9 ± 2.0	25.4 ± 0.4	26.3 ± 0.4	26.9 ± 0.7	25.8 ± 0.2
C_*org*_, mgC g^–1^ soil, 0–20 cm	28.4 ± 1.5	19.1 ± 0.2	19.7 ± 0.3	21.5 ± 0.5	20.9 ± 0.3
C_*org*_, in profile, t ha^–1^	205 ± 3.5	148 ± 1.6	154 ± 2.0	171 ± 2.3	168 ± 2.1
C_mic_, mg C g^–1^ soil, 0–20 cm	0.825 ± 0.004	0.308 ± 0.002	0.290 ± 0.001	0.480 ± 0.003	0.383 ± 0.003
C_mic_/C_*org*_, %	2.90	1.61	1.47	2.23	1.83

1, Con; 2, Min (NPK); 3, Org (manure + straw + green manure); 4, Org + Min (manure + NPK). Data are statistically significant (*P* < 0.05).

*Fertiliser doses are provided based on 1 ha of area.

**TABLE 4:** Content and reserves of organic matter and carbon pool in the albic retisols of Ukraine (x ± SD, *n* = 15).

Treatment	Natural ecosystem	Agroecosystem*			
		**1**	**2**	**3**	**4**
Humus, %	2.0 ± 0.03	1.0 ± 0.01	1.1 ± 0.01	1.2 ± 0.01	1.2 ± 0.01
Humus in profile, t ha^–1^	153 ± 3.6	78 ± 2.1	84 ± 2.3	106 ± 2.5	108 ± 2.5
SOM in profile, t ha^–1^	178 ± 3.9	89 ± 1.3	98 ± 1.6	123 ± 1.4	119 ± 1.3
C_tot_, mg C g^–1^ soil, 0–20 cm	14.3 ± 1.3	7.7 ± 0.1	8.1 ± 0.2	9.8 ± 0.3	9.5 ± 0.3
C_*org*_, mgC g^–1^ soil, 0–20 cm	11.6 ± 0.9	5.3 ± 0.2	5.8 ± 0.2	7.0 ± 0.4	6.5 ± 0.3
C_*org*_, in profile, t ha^–1^	89 ± 2.1	44 ± 1.5	49 ± 1.4	62 ± 1.6	58 ± 1.5
C_mic_, mg C g^–1^ soil, 0–20 cm	0.113 ± 0.003	0.054 ± 0.001	0.061 ± 0.002	0.075 ± 0.003	0.068 ± 0.002
C_mic_/C_*org*_, %	0.97	1.02	1.05	1.07	1.05

1, Con; 2, Min (NPK); 3, Org (manure); 4, Org + Min (manure + NPK). Data are statistically significant (*P* < 0.05).

*Fertiliser doses are provided based on 1 ha of area.

Utilising soils for agricultural production has led to changes in soil processes and properties, evidenced by a significant loss of OM in soils of agro-ecosystems. In arable soils, the content and reserves of humus and SOM decreased by an average of 1.5–2 times. The most significant loss of humus reserves in the soil profile was in albic retisols (1.96–1.44 times) and the smallest in chernic phaeozems (1.27–1.81 times). These results are shown in Tables 3, 4, respectively. Regarding C_org_, the average losses in the soil profile for albic retisols, greyzemic phaeozem, and chernic phaeozems were 60, 71, and 78 t ha^–1^, respectively. This is due to the imbalance of the input and removal of OM from the soil in the agro-ecosystem with the harvest and the activation of mineralisation processes. It was established that, in arable soils, the content of C_tot_ decreased by 1.3–1.6 times and that of C_org_ by 1.5–1.9 times. At the same time, the fraction of C_org_ in the total soil carbon pool in the agro-ecosystem is 69–75%, and in the soils of the natural ecosystem, 81–92%.

Microbial communities in soils participate in the processes of accumulation and decomposition of plant organic carbon (Miltner et al., 2012; Demyanyuk et al., 2019; Symochko, 2020; Rodrigues et al., 2023), but for a long time, it has remained a challenge to directly connect the microbiological activity of the soil with its ability to absorb C in the soil. Up to 5% of the total amount of SOM is represented by microbial biomass, and it is a more sensitive indicator of changes in soil conditions than direct analysis of C_org_ (Leita et al., 1999). Compared to their natural counterparts, the loss of C_org_ in arable soils was 29-47% due to a significant decrease (43–56%) in the C_mic_ content. This can be attributed to the application of agricultural fertilisers that cause an imbalance in the microbial community of the soil, reduce microbial productivity, and activate the processes of mineralisation of OM (Demyanyuk et al., 2019; Symochko, 2020). In contrast, the soil of natural ecosystems is characterised by a high content of C_mic_ as part of C_org_, which indicates high microbial productivity and fixation of organic substances in the biomass of microorganisms. In the natural ecosystem, the share of C_mic_ in the composition of C_org_ is 0.97% in albic retisols, 1.90% in greyzemic phaeozems (Table 5), and 2.90% in chernic phaeozems. However, for the soils of the agro-ecosystem, a decrease in the content of C_mic_ in the composition of SOM was recorded on average to the level of 1.47–1.52% in greyzemic phaeozems and 1.61–2.23% in chernic phaeozems.

**TABLE 5:** Content and reserves of organic matter and carbon pool in the greyzemic phaeozems soils of Ukraine (x ± SD, *n* = 15).

Treatment	Natural ecosystem	Agroecosystem*		
		**1**	**2**	**3**
Humus, %	2.8 ± 0.03	1.8 ± 0.001	2.0 ± 0.001	2.1 ± 0.001
Humus in profile, t ha^–1^	198 ± 4.1	124 ± 2.2	143 ± 2.6	160 ± 2.4
SOM in profile, t ha^–1^	224 ± 3.9	137 ± 1.6	161 ± 2.0	185 ± 1.9
C_tot_, mg C g^–1^ soil, 0–20 cm	18.4 ± 1.5	11.2 ± 0.8	13.2 ± 1.1	13.5 ± 1.0
C_*org*_, mgC g^–1^ soil, 0–20 cm	16.2 ± 1.1	8.2 ± 0.8	9.5 ± 0.8	10.1 ± 1.0
C_*org*_, in profile, t ha^–1^	112 ± 2.3	68 ± 1.5	79 ± 1.7	92 ± 2.0
C_mic_, mg C g^–1^ soil, 0–20 cm	0.308 ± 0.003	0.125 ± 0.002	0.132 ± 0.002	0.148 ± 0.003
C_mic_/C_*org*_, %	1.90	1.52	1.39	1.47

1, Con; 2, Min (NPK); 3, Org + Min (manure + green manure + straw + NPK). Data are statistically significant (*P* < 0.05).

*Fertiliser doses are provided based on 1 ha of area.

**TABLE 6:** Potential cellulolytic activity and CO_2_ emissions in natural and agricultural ecosystems in albic retisols (x ± SD, *n* = 5).

Parameter	Natural ecosystem	Agroecosystem			
		**1**	**2**	**3**	**4**
Cellulolytic microorganisms, × 10^3^ CFU g^–1^ d.s.	4.2 ± 0.7	12.0 ± 1.3	29.3 ± 2.6	55.4 ± 2.9	37.9 ± 2.5
Cellulolytic activity of soil, %	20.8 ± 1.3	24.5 ± 1.1	37.1 ± 1.4	39.9 ± 1.8	36.9 ± 1.3
Emission CO_2_, mg CO_2_ kg^–1^ of soils per day	14.05 ± 0.6	25.1 ± 1.3	33.4 ± 1.6	34.0 ± 1.6	30.1 ± 1.4

1, Con; 2, Min (NPK); 3, Org (manure); 4, Org + Min (manure + NPK). Data are statistically significant (*P* < 0.05).

This study evaluated the concentration of C_mic_ in the SOM of agro-ecosystems under different fertilisation systems while accounting for the rapid response of microbial biomass to changes in environmental factors and anthropogenic impact. The findings revealed that long-term and consistent application of mineral fertilisers in chernic phaeozems led to an 8–21% decrease in C_org_ content. Similarly, in greyzemic phaeozems, a 12–33% reduction was observed, and in albic retisols, the decrease ranged from 6 to 38%. Conversely, with the systematic introduction of organic and organo-mineral fertilisers in albic retisols and greyzemic phaeozems, an average increase in the C_org_ content by 6–9% was noted. In the case of chernic phaeozems, the increase was more significant, reaching 11–12%. This is confirmed by the ratio of C_mic_ to C_*org*_, which are indicators of the ecological state of the soil and the availability of C_*org*_.

The part of microbial biomass carbon in the gross SOM content (C_mic_/C_*org*_) is an important ecological and physiological parameter of the microbial community, which reflects its trophic level. The C_mic_/C_*org*_ ratio is also an indicator of the presence of available soil carbon for microorganisms, and the narrowing of this ratio in the soil indicates the stability of the organic substrate or the presence of conditions that prevent the growth of microorganisms. In this study, the fraction of carbon in microbial biomass increased by an average of 15% when fertilisers were applied. In agro-ecosystem soils, the most significant C fraction in microbial biomass from the carbon of organic compounds was noted under organic and organo-mineral fertilisation systems; for chernic phaeozems, values were 1.6 and 1.8%, for greyzemic phaeozems 2.7 and 3.1%, and for albic retisols 3.2 and 3.3%, respectively. Compared to mineral fertilisers, more favourable conditions for soil microbiota development and microbial biomass accumulation were formed when organic fertilisers were applied. Moreover, under the organo-mineral fertilisation system, the ratio of C_mic_/C_*org*_ was at a maximum at 3.5, 3.1, and 1.9%, respectively, in albic retisols, greyzemic phaeozems, and chernic phaeozems, which indicates a sufficient supply of nutrients to soil microorganisms, active decomposition of carbon compounds, and immobilisation of carbon in its biomass. Therefore, due to the prolonged use of soils with and without fertilisers, there is a significant decrease in the total biomass of microorganisms and the ratio of C_mic_/C_org_, indicating a decrease in the specific proportion of C_mic_ in the overall C_org_ content of the soil.

Low values of C_mic_/C_org_ in agro-ecosystem soils indicate a predominance of mineralisation processes over SOM humification. Conversely, high values of C_mic_/C_org_ in natural soil biocoenosis indicate a predominance of C_mic_ synthesis processes over its degradation. The activity of soil microorganisms plays a crucial role in SOM decomposition. Notably, cellulose is one of the main biopolymers present in SOM and plant residues, and the intensity of its mineralisation depends on the abundance and activity of cellulolytic microorganisms. Indeed, the results showed a significant difference in the values of cellulose degradation activity and cellulolytic microorganisms abundance in natural ecosystems and agro-ecosystems (Tables 7, 8). A significant difference of 2.1–8.0 times was found in the abundance of aerobic cellulolytic microorganisms and 1.3–3.3 times in the investigated soils’ potential cellulolytic activity. In addition, the statistical analysis revealed a strong correlation between cellulose degradation activity and the abundance of cellulolytic microorganisms (correlation coefficient *r* > 0.7). A significant difference in the abundance of cellulolytic microorganisms was found depending on the soil type. For example, the lowest content of cellulolytic microorganisms occurred in the albic retisols (Table 6), and a natural ecosystem with albic retisols was characterised by a low level of cellulolytic activity of 20.8 ± 1.3%.

**TABLE 7:** Potential cellulolytic activity and CO_2_ emissions in natural and agricultural ecosystems in greyzemic phaeozems (x ± SD, *n* = 5).

Parameter	Natural ecosystem	Agroecosystem		
		**1**	**2**	**3**
Cellulolytic microorganisms, × 10^3^ CFU g^–1^ d.s.	22.9 ± 1.2	39.5 ± 1.4	60.8 ± 2.1	66.3 ± 2.4
Cellulolytic activity of soil, %	29.3 ± 1.3	33.2 ± 1.2	40.6 ± 1.4	42.8 ± 1.4
Emission CO_2_, mg CO_2_ kg^–1^ of soils per day	26.7 ± 0.7	26.4 ± 1.1	39.4. ± 1.5	44.4 ± 1.8

1, Con; 2, Min (NPK); 3, Org + Min (manure + green manure + straw + NPK). Data are statistically significant (*P* < 0.05).

**TABLE 8:** Potential cellulolytic activity and CO_2_ emissions in natural and agricultural ecosystems in chernic phaeozems (x ± SD, *n* = 5).

Parameter	Natural ecosystem	Agroecosystem			
		**1**	**2**	**3**	**4**
Cellulolytic microorganisms, × 10^3^ CFU g^–1^ d.s.	32.3 ± 1.1	55.0 ± 2.1	61.7 ± 1.9	79.9 ± 2.3	70.1 ± 2.3
Cellulolytic activity of soil, %	12.5 ± 0.8	35.5 ± 1.4	39.0 ± 1.3	47.6 ± 1.6	44.9 ± 1.3
Emission CO_2_, mg CO_2_ kg^–1^ of soils per day	29.5 ± 0.7	15.8 ± 1.1	21.4 ± 1.3	28.2 ± 1.3	25.3 ± 1.3

1, Con; 2, Min (NPK); 3, Org (manure + straw + green manure); 4, Org + Min (manure + NPK). Data are statistically significant (*P* < 0.05).

Results further revealed that an abundance of cellulose-degrading microorganisms was characteristic of chernic phaeozems with high SOM content. This surpassed the other investigated soil types by 1.4 times for greyzemic phaeozems and 7.8 times for albic retisols. Among the studied soil types, high values of CO_2_ emissions were observed in chernic phaeozems soil with high OM content. The activity of heterotrophic microorganisms involved in the mineralisation of OM accounts for approximately 70% of CO_2_ emissions from the soil, and agro-ecosystems have the most dynamic OM balance. Carbon loss in arable soils due to careless and irrational use transforms agro-ecosystems into significant sources of CO_2_ emissions to the atmosphere. Additionally, it has been established that high levels of CO_2_ emissions are characteristic of arable soils, where agricultural practices, such as fertiliser application, especially mineral fertilisers, stimulate SOM decomposition and CO_2_ release. However, the long-term and systematic application of mineral fertilisers creates a stressful and unfavourable environment for the microbiota, leading to the development and activation of mycelial organisms, including a significant number of cellulose-degrading microorganisms.

The stimulating effect of mineral fertilisers on the development of cellulolytic microorganisms and their activity may result from the redistribution within the microbial community structure toward more resistant organisms, such as micromycetes and actinomycetes (Francioli et al., 2016; Symochko, 2020). In chernic phaeozems, cellulolytic activity (Table 7) was significantly higher in agro-ecosystems (47.6–35.5%) in comparison with natural ecosystems (12.5%).

**FIGURE 1:**
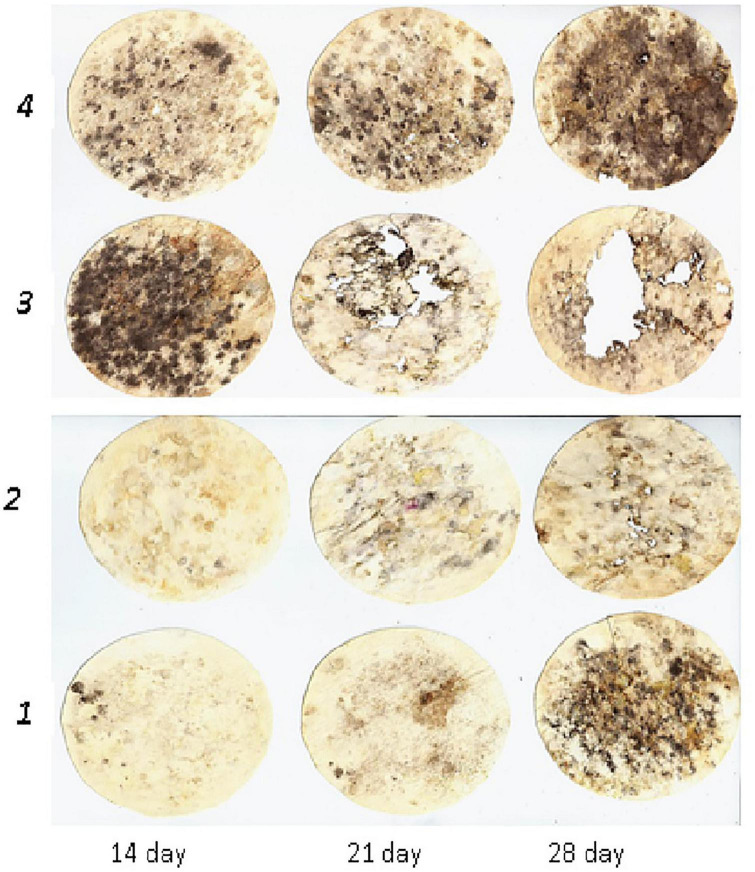
Dynamics of decomposition of cellulose substrate in chernic phaeozems: 1, Con; 2, Min (NPK); 3, Org (manure + straw + green manure); 4, Org + Min (manure + NPK).

**FIGURE 2:**
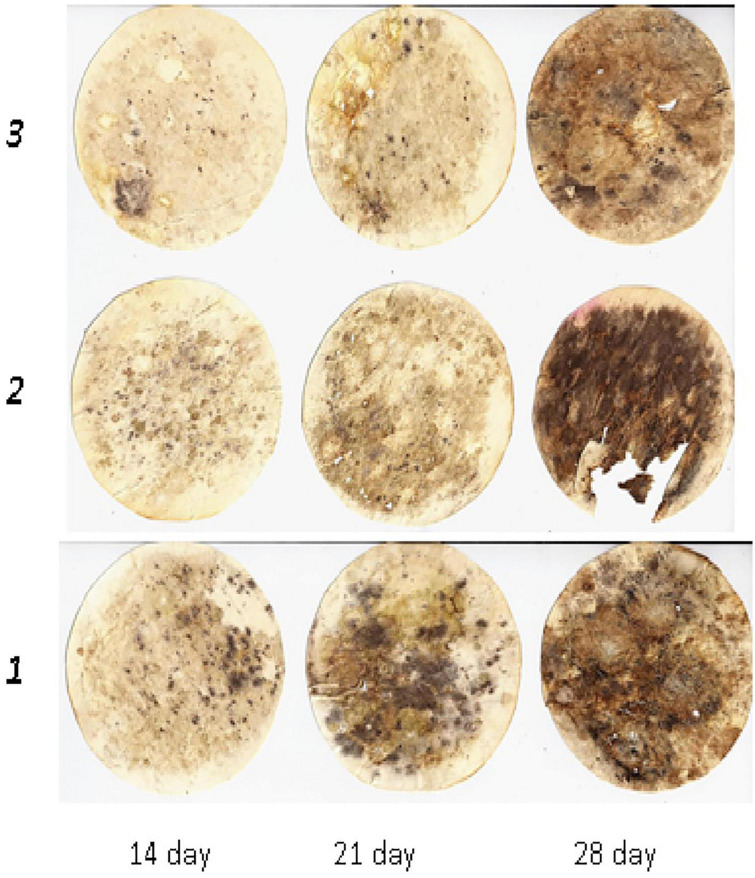
Dynamics of cellulose substrate decomposition in greyzemic phaeozems: 1, Con; 2, Min (NPK); 3, Org + Min (manure + green manure + straw + NPK).

**FIGURE 3:**
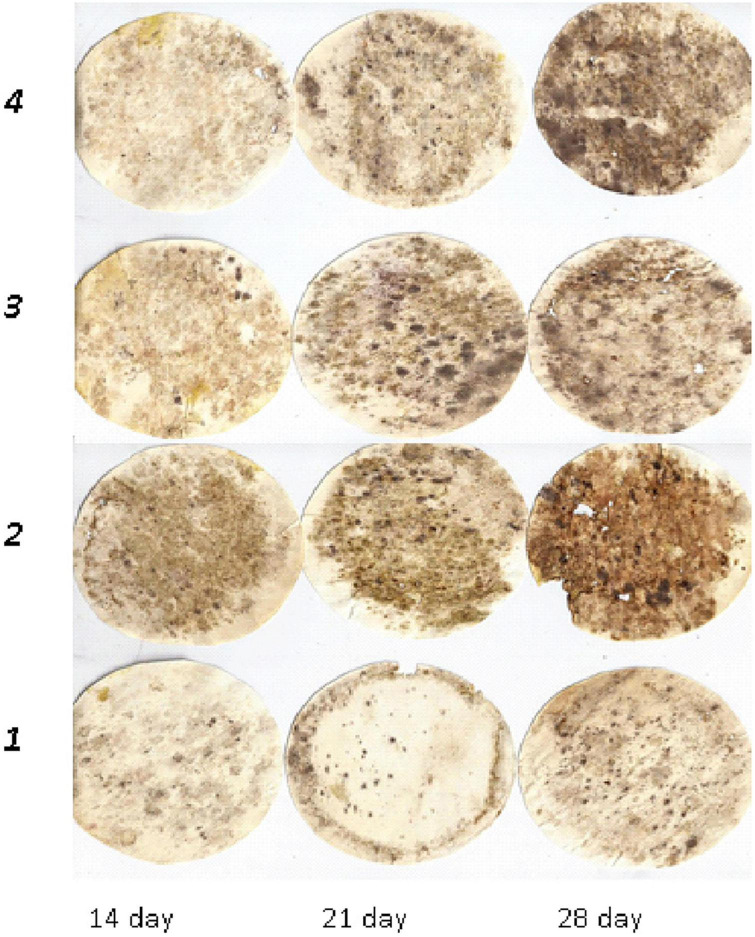
Dynamics of cellulose substrate decomposition in albic retisols: 1, Con; 2, Min (NPK); 3, Org (manure); 4, Org + Min (manure + NPK).

Figures 1–3 depict the dynamics of cellulose substrate decomposition in different types of arable soils under various fertilisation systems. Various types of fertilisers had distinct and notable impacts on the activation of cellulolytic microorganisms, which play a key role in breaking down plant-based biopolymers (Tang et al., 2021). Notably, these microorganisms thrived in scenarios where organic fertilisers, such as manure and cover crops, were applied. These organic fertilisers contain substantial amounts of cellulose polysaccharides, fuelling the vigorous development of cellulolytic microorganisms. For example, active cellulose decomposition was observed in the variant with long-term application of fresh organic substrate (cover crop). This process was most active, regardless of soil type, in variants with organic and organo-mineral fertilisation systems. In addition, applying mineral fertilisers in combination with organic fertilisers promoted the activation of cellulose decomposition and cellulolytic activity increased by 2.8 times. In the presence of crop residues, it increased by 4.5 times. In the unfertilised variants, the activity of cellulose substrate decomposition was low compared to other experimental variants. Specifically, it was 12.0% in the sod-podzolic soil, 39.5% in the dark grey soil, and 55.0% in the chernic phaeozems after 1 month of exposure. This corresponds to a weak degree of cellulose biopolymer decomposition intensity. In these variants, there is a deficiency of organic substrate for soil microbiota, and organic residues and plant litter cannot compensate for the processes of humus degradation and mineralisation. In chernic phaeozems (Figure 1), high cellulolytic activity was observed in the soil of variants with simultaneous application of manure, straw, and cover crop (79.9%), as well as with the combined application of mineral fertilisers and manure (71.3%).

This was also observed in greyzemic phaeozems (Figure 2) and albic retisols (Figure 3), where the application of mineral fertilisers contributed to the intensification of decomposition processes in the soil, giving them an advantage over other experimental variants by 8–15%.

Applying mineral fertilisers in combination with different organic substances leads to a shift in the microbial transformation of plant substrates toward complete mineralisation and the release of carbon in the form of CO_2_ into the atmosphere. This has been confirmed by results conducted on various soil types (Sedlár et al., 2023). For example, on chernic phaeozems, adding straw with a green mass of cover crops, manure, and mineral fertilisers stimulated the process of CO_2_ release, exceeding that of the control and mineral fertiliser system treatments by an average of 29.5–50.2%. A similar situation was observed in greyzemic phaeozems, where the combined application of wheat straw with cover crops, manure, and mineral fertilisers increased the activity of CO_2_ release by 39.8–48.1%.

It is known that the decomposition of the siderate biomass, rich in carbohydrates and proteins, occurs much faster than straw, which has a higher content of aromatic compounds of phenolic nature in its composition and a wide C:N ratio (Getino-Álvarez et al., 2023). The presence of straw in combination with siderates has a notable effect: it retards the decomposition of the green siderate mass, leading to favourable conditions for humus accumulation. Consequently, under these conditions, CO2 emissions from chernic phaeozems decrease by 13–16%, in contrast to scenarios where manure, straw, siderates, and mineral fertilisers are all applied simultaneously.

## 4 Conclusion

The decomposition of OM in soil is a crucial global process, as an increase in the carbon mineralisation rate is expected in response to predicted temperature rise and unsustainable use of soil resources. In the leached soils of Ukraine, the OM content has been determined at levels of 178 t ha^–1^ in albic retisols, 224 t ha^–1^ in greyzemic phaeozems, and 410 t ha^–1^ in chernic phaeozems, which is 1.3–1.7 times higher than in agro-ecosystem soils. A high content of C_mic_ characterises the soils of natural ecosystems.

The involvement of soils in agricultural use has led to changes in soil processes and properties, as evidenced by significant loss of OM in agro-ecosystem soils and the intensification of degradation processes. In this study, the most significant loss of humus reserves in the soil profile was observed in albic retisols, ranging from 1.96 to 1.44 times, while the lowest loss occurred in chernic phaeozems, ranging from 1.27 to 1.81 times. Regarding C_org_, the losses in the soil profile were 60 t ha^–1^ in albic retisols, 71 t ha^–1^ in greyzemic phaeozems, and 78 t ha^–1^ in chernic phaeozems. Furthermore, the long-term and systematic application of mineral fertilisers has led to an 8–21% decrease in C_org_ content in chernic phaeozems, 12–33% in dark grey soils, and 6–38% in albic retisols. However, the systematic application of organic and organo-mineral fertilisers in albic retisols and greyzemic phaeozems resulted in an average increase of C_org_ content by 6–9%, while it increased by 11–12% in chernic phaeozems. High numbers of cellulose-degrading microorganisms were found in chernic phaeozems with a high OM content in the soil, in compare with other types of investigated soils. Among the studied soil types, high CO_2_ emissions were observed in chernic phaeozems.

## Data availability statement

The original contributions presented in this study are included in this article, further inquiries can be directed to the corresponding author.

## Author contributions

LS: Conceptualization, Investigation, Methodology, Supervision, Validation, Writing – original draft. OD: Formal analysis, Methodology, Software, Supervision, Validation, Writing – original draft. VC: Funding acquisition, Visualization, Writing – review and editing. LD: Funding acquisition, Visualization, Writing – review and editing.
